# Corrigendum: Phosphines are ribonucleotide reductase reductants that act via C-terminal cysteines similar to thioredoxins and glutaredoxins

**DOI:** 10.1038/srep45880

**Published:** 2017-04-07

**Authors:** Vladimir Domkin, Andrei Chabes

Scientific Reports 4: Article number: 5539; 10.1038/srep05539 published online: 07 02 2014; updated: 04 07 2017.

This Article contains a typographical error in the Methods section under subheading ‘**Ribonucleotide Reductase Assay’**.

“Supernatants were loaded onto pre-washed 2 mL Dowex AG 1X8, 200–400 in [H^+^]-form columns prepared by replacing the Sephadex in NAP-10 columns (Amersham Bioscience) with 2 mL of Dowex from Bio-Rad pre-packed columns”.

should read:

“Supernatants were loaded onto pre-washed 2 mL Dowex AG 50W-X8, 200–400 in [H^+^]-form columns prepared by replacing the Sephadex in NAP-10 columns (Amersham Bioscience) with 2 mL of Dowex from Bio-Rad pre-packed columns”.

